# Respiratory Muscle Training Improves Functional Outcomes and Reduces Fatigue in Patients with Myasthenia Gravis: A Single-Center Hospital-Based Prospective Study

**DOI:** 10.1155/2020/2923907

**Published:** 2020-03-19

**Authors:** Che-Wei Hsu, Hui-Chen Lin, Wan-Chen Tsai, Yun-Ru Lai, Chih-Cheng Huang, Yu-Jih Su, Ben-Chung Cheng, Mao-Chang Su, Wei-Che Lin, Chia-Ling Chang, Wen-Neng Chang, Meng-Chih Lin, Cheng-Hsien Lu, Nai-Wen Tsai

**Affiliations:** ^1^Department of Neurology, Chang Gung Memorial Hospital-Kaohsiung Medical Center, Chang Gung University College of Medicine, Kaohsiung, Taiwan; ^2^Department of Biological Science, National Sun Yat-sen University, Kaohsiung, Taiwan; ^3^Department of Rheumatology, Chang Gung Memorial Hospital-Kaohsiung Medical Center, Chang Gung University College of Medicine, Kaohsiung, Taiwan; ^4^Department of Nephrology, Chang Gung Memorial Hospital-Kaohsiung Medical Center, Chang Gung University College of Medicine, Kaohsiung, Taiwan; ^5^Department of Chest, Chang Gung Memorial Hospital-Kaohsiung Medical Center, Chang Gung University College of Medicine, Kaohsiung, Taiwan; ^6^Department of Radiology, Chang Gung Memorial Hospital-Kaohsiung Medical Center, Chang Gung University College of Medicine, Kaohsiung, Taiwan; ^7^Department of Respiratory Therapy, Chang Gung Memorial Hospital-Kaohsiung Medical Center, Chang Gung University College of Medicine, Kaohsiung, Taiwan

## Abstract

**Background:**

Myasthenia gravis (MG) is an immune-mediated disorder characterized by muscle fatigue and fluctuating weakness. Impairment in respiratory strength and endurance has been described in patients with generalized MG. We tested the hypothesis that respiratory muscle training (RMT) can improve functional outcomes and reduce fatigue in patients with MG.

**Methods:**

Eighteen patients with mild to moderate MG participated in this study. The training group underwent home-based RMT three times a week for 12 weeks. Sixteen patients with MG without RMT were enrolled as a disease control group. Lung function, autonomic testing, Multidimensional Fatigue Symptom Inventory-Short Form (MFSI-SF), and functional outcome measurement by using quantitative myasthenia gravis (QMG) score and myasthenia gravis composite (MGC) scale were measured before and after the 12-week RMT.

**Results:**

The 12-week RMT significantly increased forced vital capacity (FVC) from 77.9 ± 12.6% to 83.8 ± 17.7% (*p* = 0.03), forced expiratory volume in one second (FEV1) from 75.2 ± 18.3% to 83.3 ± 19.0% (*p* = 0.03), forced expiratory volume in one second (FEV1) from 75.2 ± 18.3% to 83.3 ± 19.0% (*p* = 0.03), forced expiratory volume in one second (FEV1) from 75.2 ± 18.3% to 83.3 ± 19.0% (*p* = 0.03), forced expiratory volume in one second (FEV1) from 75.2 ± 18.3% to 83.3 ± 19.0% (*p* = 0.03), forced expiratory volume in one second (FEV1) from 75.2 ± 18.3% to 83.3 ± 19.0% (*p* = 0.03), forced expiratory volume in one second (FEV1) from 75.2 ± 18.3% to 83.3 ± 19.0% (

**Conclusion:**

The home-based RMT is an effective pulmonary function training for MG patients. The RMT can not only improve short-term outcomes but also reduce fatigue in patients with mild to moderate generalized MG.

## 1. Introduction

Myasthenia gravis (MG) is an immune-mediated neuromuscular junction disorder characterized by fluctuating muscle weakness and easy fatigability. In most cases, autoantibodies against the acetylcholine receptor can be found [1]. Impairment in respiratory strength and endurance has been described in patients with generalized MG [2]. Respiratory muscle dysfunction can further deteriorate patients' physical fitness and even increase the risk of respiratory failure as the characteristic feature of myasthenic crisis [3]. Improvement of respiratory muscle function is therefore an important goal in MG therapy.

The Myasthenia Gravis Foundation of America Clinical Classification divides MG into 5 main classes according to signs and symptoms [4]. Class I is defined as patients with any ocular muscle weakness and all other muscle strength as normal. Classes II to IV are defined as patients with mild to severe muscle weakness affecting other than ocular muscles, respectively. Class V is defined by the need for intubation, with or without mechanical ventilation, except when used during routine postoperative management. The effect of RMT may be performed safely and effectively in mild to moderate MG patients (classes II and III) with impairment of respiratory function [5, 6].

A previous study demonstrates that home-based respiratory muscle training (RMT) combined with breathing retraining in patients with generalized MG leads to improvements in respiratory muscle strength, chest wall mobility, and respiratory muscle endurance but does not appear to improve lung function [5, 7]. Lung function parameters such as vital capacity (VC), forced expiratory volume in one second (FEV1), and maximal expiratory pressure (MEP) are based on short maneuvers requiring maximal effort. These abilities are usually not reduced in patients with mild to moderate MG. Fatigue and weakness of respiratory muscles in MG patients are responsible for dyspnea, reduced exercise tolerance, and increased risk of respiratory failure. Therefore, improved respiratory endurance is even more important than improvement of lung function parameters in MG patients [8].

To our knowledge, few studies have demonstrated that RMT is associated with effects of functional outcome and fatigue in MG patients. The present study is therefore aimed at assessing the training effects of RMT on MG symptoms and pulmonary function in patients with mild to moderate MG. We investigated whether the RMT not only enhances the functional outcome but also reduces the fatigue in patients with MG.

## 2. Materials and Methods

### 2.1. Participants

This single-center hospital-based prospective study enrolled participants with mild to moderate generalized MG (classes II to III according to MGFA classification) [4], recruited consecutively from Chang Gung Memorial Hospital-Kaohsiung, a tertiary medical center and the main referral hospital in southern Taiwan. A diagnosis of MG is based on clinical features with serial examinations in terms of electromyography, serum autoantibodies, chest CT scan, and effect of cholinesterase inhibitors [9].

Exclusion criteria included the following: (1) presence of significant diseases (class III of MGFA classification) who would not be able to complete the training; (2) MG patients with ocular symptoms only (class I of MGFA classification); (3) MG patients in the state of myasthenic crisis; (4) presence of underlying malignancy or hematological disorders; and (5) history major systemic disease, such as end-stage renal disease, liver cirrhosis, and heart failure.

For a statistical power of 80% and the significance level of 5%, a sample size of 18 participants was calculated to determine a 15% change in myasthenia score improvement [5]. To avoid the influence of age, sex, and body mass index on the pulmonary function [10, 11], sixteen age-, sex-, and BMI-matched MG patients who were not willing to undergo RMT were included as disease controls. All participants have signed an informed consent form, and the study was conducted in accordance with the Declaration of Helsinki and approved by the hospital's Institutional Review Committees on Human Research (IRB 105-5274C). The Transparent Reporting of Evaluations with Nonrandomized Designs (TREND) statements were used to report all the different steps of the interventions utilized in this study [12, 13].

### 2.2. Respiratory Muscle Training (RMT)

**Figure 1:**
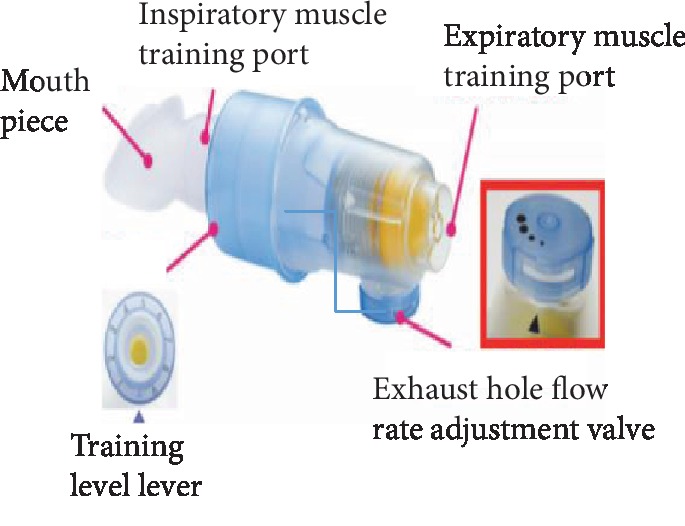
Dofin Breathing Trainer: the device for respiratory muscle training.

Despite normal spirometric values, patients with generalized MG often present a characteristic pattern with a decreasing respiratory muscle strength [14] and reduced respiratory muscle endurance [2]. Due to the fatigue-prone nature of the repetitive exercise for MG patients, we choose the interval-based RMT method. The protocol of RMT was modified from previous studies as follows [15, 16]. RMT was performed by using the Dofin Breathing Trainer, a handheld pressure threshold device (Figure 1). The device can be calibrated up to a pressure range of 5-39 cmH_2_O for inspiratory muscle training and 4-33 cmH_2_O for expiratory muscle training. RMT was applied to generate both expiratory force for cough function and inspiratory muscle straining for the lung ventilation impairments. Patients with respiratory muscle weakness received an inspiratory muscle training from 30% to 60% of the maximum inspiratory pressures (MIP) through a respiratory trainer for two sets of 30 breaths or 6 sets of 10 repetitions. For patients with swallowing disturbance, the expiratory muscle strengthening training commences from 15% to 75% of the threshold load of an individual's maximum expiratory pressures (MEP), 5 sets of 5 repetitions with one minute of rest between sets. The training resistance was adjusted accordingly, with one or two minutes of rest between sets. RMT was conducted by an experienced respiratory therapist at the time of enrollment, and it was ensured that the participants were familiar with the device. All the participants were trained for 30 min/day twice per day, for at least 5 days a week for 12 weeks, and were monitored by making a phone call to them once a week to check the compliance of RMT at home.

### 2.3. Pulmonary Function Testing

The pulmonary function testing of every participant included forced vital capacity (FVC), forced expiratory volume in one second (FEV1), and FEV1/FVC indexes using spirometry without exposure to a bronchodilator. In respiratory strengths, respiratory pressure was measured under static conditions, with MIP and MEP at a total lung capacity. Pulmonary function values were based on the best of three efforts. The procedure of spirometry completely followed the guidelines of the American Thoracic Society [17], and the results of the pulmonary function test are classified into three patterns as follows [18]: (i) obstructive pattern, which was defined as FEV1/FVC < 0.7; (ii) restrictive pattern, which was defined as FEV1/FVC ≥ 0.7 with FVC < 80%; and (iii) normal pattern, which was defined as FEV1/FVC ≥ 0.7 with FVC ≥ 80%. The six-minute walk test was used to describe walking capability among patients with MG [19]. In previous studies, the six-minute walk test was shown to be an exercise capacity test in neuromuscular diseases [19, 20], and the normal values of the mean distance have been well defined [21, 22].

### 2.4. Clinical Assessment

All subjects underwent complete neurological examinations, pulmonary function, and self-administered questionnaires upon enrollment and 12 weeks after RMT. Outcomes were measured by using the quantitative myasthenia gravis (QMG) score and myasthenia gravis composite (MGC) scale. The QMG has several items that measure endurance or fatigability, taking into account the fluctuating nature of the disease. The 13 items are as follows: ptosis, diplopia, orbicularis oculi weakness, swallowing a cup of water, speech, percent predicted forced vital capacity, grip strength (2 items), arm endurance (2 items), leg endurance (2 items), and neck flexion endurance. All items are scored from 0 (no symptoms) to 3 (severe symptoms), with a total score ranging from 0 to 39; higher scores indicate greater disease severity [23].

The MGC scale contains a total of 6 physician-evaluated items, 2 ocular items (diplopia and ptosis) from the QMG, 4 items (facial, neck, deltoids, and hip flexor strength) from the Manual Muscle Test, and 4 patient-reported items (chewing, swallowing, breathing, and speech). Items are scored using a 4-level severity assessment (normal/no symptoms to severe symptoms), with weighted point scores for each item summed to generate a total MGC score ranging from 0 (no symptoms) to 50 (maximum severity) [24]. The MGC was recommended as the primary outcome measure of choice in MG trials by the MGFA scientific board [25], and it has been subsequently used as a primary or secondary outcome in several trials [26].

### 2.5. Measurement of Fatigue

The fatigue was measured by the self-administered questionnaire Multidimensional Fatigue Symptom Inventory-Short Form (MFSI-SF). The version has been validated in Chinese population [27]. The MFSI-SF is a 30-item short form of the MFSI that yields scores only for the empirically derived subscales, each scored from 0 (not at all) to 4 (extremely). Previous research suggests that it has acceptable psychometric properties and may be used as a substitute for the MFSI when time constraints and scale length are of concern [28]. The MFSI-SF scoring for the empirically derived scales is as follows: (1) general scale; (2) physical scale; (3) emotional scale; (4) mental scale; (5) vigor scale; and (6) total score = (general + physical + emotional + mental)–vigor.

### 2.6. Statistical Analysis

Data were expressed as mean ± standard deviation (SD) or median (interquartile range (IQR)). Categorical variables were compared using chi-squared or Fisher's exact tests. Continuous variables were compared in two patient groups (the RMT group and the disease control group) by using independent *t*-tests. The data of cardiovascular autonomic function (HR_DB, Valsalva ratio, BRS_seg, and LF/HF ratio) that were not normally distributed were logarithmically transformed to improve normality for comparison. Second, changes between baseline and 12 weeks post-RMT on parameters of respiratory parameters, fatigue score, and outcome score were compared using a paired *t*-test and the Wilcoxon signed-rank test for nonparametric data. Furthermore, repeated-measure ANOVA was used to compare parameters and functional scores at two different time points (enrollment and 12 weeks follow-up). Statistical significance was set at *p* < 0.05. All statistical analyses were conducted using the SAS software version 9.1 (SAS Statistical Institute, Cary, NC, USA).

## 3. Results

### 3.1. Baseline Characteristics between the RMT Group and the Disease Control Group

**Figure 2:**
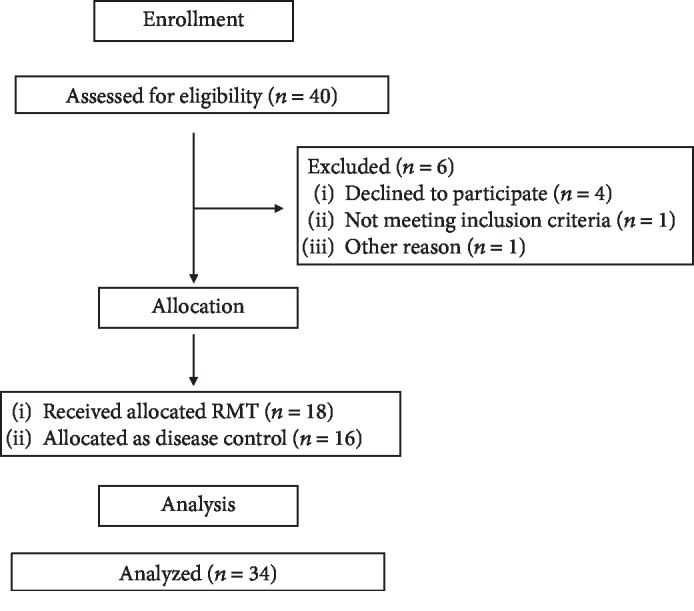
Flow diagram of the clinical intervention.

The flow diagram with the enrolment of the study is shown in Figure 2. Forty individuals were recruited, and six were excluded. Consequently, thirty-four participants (18 cases in the RMT group and 16 participants in the disease control group) are enrolled in this study. They have been diagnosed with MG for 1–25 years.  Table 1 shows the baseline characteristics between groups of the RMT patients and disease controls. There was no significant difference between the RMT group and the disease control group in terms of age, sex, body weight, body high, duration of disease, cardiovascular autonomic function, pulmonary function parameters, or MGFA classification.

### 3.2. Change of Pulmonary Function after RMT

The changes of pulmonary function parameters during the study period are shown in Table 2. The parameters of pulmonary function, including FVC (77.9 ± 12.6% to 83.8 ± 17.7%, *p* = 0.03), FEV1 (75.2 ± 18.3% to 83.3 ± 19.0%, *p* = 0.002), and 6-minute walking distance (403.4 ± 72.2 m to 466.1 ± 68.5 m, *p* = 0.003), all significantly increased after 12-week training in the RMT group. On the contrary, the pulmonary function parameter in the two measures was not significantly different in the control group.

### 3.3. Comparison of MG Outcome Scale and Fatigue Score before and after RMT

**Figure 3:**
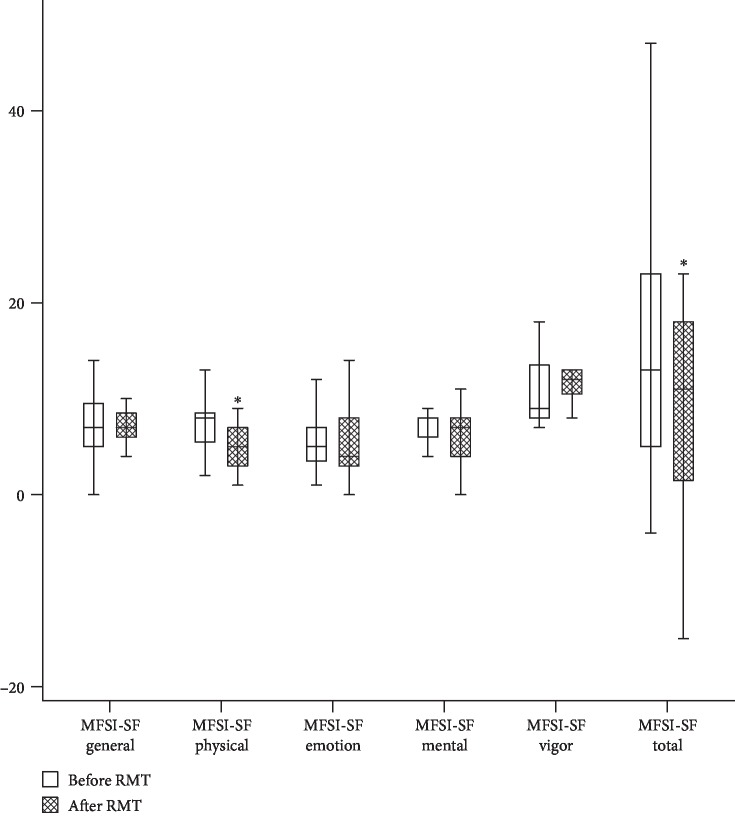
Comparison of MFSI-SF scoring between baseline and after RMT in patients with myasthenia gravis. ^∗^*p* < 0.05 compared to the baseline.

Table 3 shows the results of MG outcomes and fatigue score before and after RMT. The QMG score (*p* = 0.02) and MGC score (*p* = 0.05) significantly reduced after RMT when compared to baseline, which means the MG outcomes in the RMT group were improving. However, the disease control group had similar scores at baseline and during follow-up.  Figure 3 shows the comparison of MFSI-SF scoring between baseline and after RMT. The physical subscale of MFSI-SF was significantly lower than the baseline data for the RMT group during follow-up (*p* = 0.02). The total score of MFSI-SF significantly reduced after RMT when compared to the baseline score (*p* = 0.04). On the contrary, the fatigue scores in the two measures (baseline and follow-up) were not significantly different in the disease control group.

## 4. Discussion

The present study examined the RMT effects on pulmonary function, MG outcomes, and fatigue in MG patients. There were three main findings in this study. First, a 12-week home-based RMT may improve the pulmonary function (FVC and FVE1) and increase the 6-minute walking distance in MG patients. Second, adjunctive RMT to conventional drug treatment may enhance the short-term functional outcomes in patients with mild to moderate MG. Third, the RMT can reduce fatigue in patients with MG, especially in the physical domain.

Previous studies have inconsistent results for RMT in MG patients [7, 8, 29], probably because of different methodology and heterogeneity of patient groups, and there is no standard respiratory muscle training protocol for MG. Weiner et al. [29] demonstrated that 3 months of inspiratory muscle strength training performed 6 times per week significantly improved vital capacity and FEV1 in moderate to severe MG patients. Our research further demonstrated that RMT enhances the walking distance of patients with generalized MG. However, Fregonezi et al. [7] state that using the interval-based inspiratory muscle training 3 times a week for 8 weeks did not show any changes in lung function in MG patients. A recent study shows that long-term (thirteen months) respiratory muscle endurance training significantly increased respiratory endurance measured as time until exhaustion (*T*_lim_) to 412% of the baseline in MG patients [6]. This can be explained by the specificity of the training of different types of RMT [30]. Respiratory muscle training mainly improves maximum strength, while respiratory muscle endurance training improves endurance but not maximum force. Moreover, all our patients perceived a benefit from the RMT in terms of improved respiratory endurance and reduced fatigue symptoms. None of them reported any adverse effects and all participants agreed to continue the training study.

The MGC and QMG are outcome measures used in clinical trials and everyday practice in MG patients [31]. Our results showed that those MG patients receiving RMT had a significant improvement not only in lung function but also in functional outcomes. The main improvement items in QMG include forced vital capacity, swallowing, and speech following counting aloud 1-50. The main improvement items in MGC include swallowing and breathing. The effect of RMT in patients with MG has been shown in several previous reports [5, 6]. Weakness and fatigue of respiratory muscles are responsible for dyspnea and reduced exercise tolerance and thus can compromise quality of life. The improvement was seen not only in respiratory muscles but also in swallowing function and speech endurance.

The other important finding in this study was the reduction in fatigue after MG patients received 3 months of RMT. To the best of our knowledge, the association between RMT and fatigue in MG patients has not been reported previously. There are many causes of fatigue, including physical, emotional, and mental domains. Our research shows that RMT reduces fatigue in MG patients, mainly physical fatigue. The prevalence of fatigue is 70% in MG patients and influenced by depressive symptoms, disease severity, female sex, and sleep debt [32]. Fatigue in myasthenia affects quality of life and can be reduced after treatment [33]. Our results suggest that adjunctive RMT to conventional drug treatment not only reduce fatigue but also improve outcomes in patients with MG. Due to the characteristic of easily getting fatigued with repetitive exercise in MG, our study protocol did not prefer daily RMT because we consider it inappropriate for MG pathophysiology. Repeated exercise may cause a loss of K^+^ ions from the contracting muscle [34] and a decrease in the gradient regulated by muscle Na^+^-K^+^-adenotriphosphatase that has been related to muscular fatigue [35]. Therefore, we suggest that the interval-based RMT method is feasible and benefits the MG patients.

This study has several limitations. First, we are unable to fully monitor the status of the home-based RMT, so the completion, execution rate, and efficacy of training may be different. Future research should require participants to keep a training diary and reflect on the sessions.

Second, the study regimen was strenuous and time-consuming, and several patients who were asked for participation in this study refused. For this reason, the sample size is small and the follow-up time is relatively short. Third, during the familiarization period and the first training unit, the motivations of patients were estimated to be high but briefly declined in some patients. This also affects the differences in training. Finally, there is a lack of control in medications administered to patients with MG, which may influence the efficacy of RMT. Despite these limitations, we believe this study is a good start for further large-scale investigations in this field. Future research will assess whether the RMT protocol should be restarted after the “rest” period or if intervention should continue.

In conclusion, our study shows that RMT can not only improve respiratory and functional outcomes for patients with MG but also reduce the fatigue. Further large-scale studies can be feasible to assess adjuvant RMT for conventional drug therapy in MG patients.

## Figures and Tables

**Table 1:** Baseline characteristics between the RMT group and the disease control group.

	RMT group (*n* = 18)	Disease controls (*n* = 16)	*p* value
Age (years)	54.2 ± 14.6	62.4 ± 12.7	0.09
Sex (female)	11	10	0.61
Body weight (kg)	65.9 ± 9.8	64.2 ± 15.5	0.73
Body high (cm)	161.2 ± 9.8	157.7 ± 9.9	0.31
Disease duration (years)	10.7 ± 10.6	7.6 ± 7.9	0.43
Thymectomy	13	8	0.66
Cardiovascular autonomic function			
HR_DB	11.7 ± 6.8	13.8 ± 9.3	0.52
Valsalva ratio	1.4 ± 0.2	1.3 ± 0.2	0.53
BRS_seq	6.7 ± 4.2	4.6 ± 2.3	0.23
LF/HF ratio	1.8 ± 2.1	2.0 ± 2.0	0.79
Pulmonary function parameters			
FVC (%)	77.9 ± 12.6	82.9 ± 19.6	0.88
FEV1 (%)	75.2 ± 18.3	79.5 ± 21.8	0.51
FEV1/FVC	78.0 ± 10.5	75.5 ± 6.8	0.07
Maximum inspiratory pressures (MIP)	98.1 ± 44.2	80.9 ± 52.2	0.27
Maximum expiratory pressures (MEP)	82.5 ± 20.5	90.9 ± 33.6	0.67
6-minute walking distance (meter)	403.4 ± 72.2	394.9 ± 106.9	0.56
MGFA classification			0.12
IIa	10	6	
IIb	4	4	
IIIa	2	2	
IIIb	2	4	

Values are expressed as mean ± SD unless otherwise indicated. MGFA: Myasthenia Gravis Foundation of America classification; RMT: respiratory muscle training.

**Table 2:** Changes of pulmonary function after RMT.

	RMT group (*n* = 18)			Disease control group (*n* = 16)		
	Baseline	Follow-up	*p* value	Baseline	Follow-up	*p* value
Cardiovascular autonomic function						
HR_DB	11.7 ± 6.8	13.6 ± 8.8	0.16	13.8 ± 9.3	12.1 ± 7.1	0.62
Valsalva ratio	1.4 ± 0.2	1.4 ± 0.2	0.64	1.3 ± 0.2	1.3 ± 0.2	0.79
BRS_seq	6.7 ± 4.2	7.2 ± 4.1	0.54	4.6 ± 2.3	4.5 ± 2.5	0.91
LF/HF ratio	1.8 ± 2.1	2.1 ± 2.0	0.64	2.0 ± 2.0	1.6 ± 2.0	0.63
Pulmonary function parameters						
FVC (%)	77.9 ± 12.6	83.8 ± 17.7	0.03^∗^	82.9 ± 19.6	85.3 ± 24.7	0.62
FEV1 (%)	75.2 ± 18.3	83.3 ± 19.0	0.002^∗^	79.5 ± 21.8	82.3 ± 25.4	0.52
FEV1/FVC	78.0 ± 10.5	81.3 ± 8.4	0.26	75.5 ± 6.8	76.8 ± 6.7	0.54
MIP	98.1 ± 44.2	105.6 ± 42.4	0.26	80.9 ± 52.2	93.6 ± 46.5	0.12
MEP	82.5 ± 20.5	91.9 ± 31.7	0.11	90.9 ± 33.6	97.3 ± 35.2	0.22
6-minute walking distance (meter)	403.4 ± 72.2	466.1 ± 68.5	0.003^∗^	394.9 ± 106.9	413.3 ± 104.2	0.39

HR_DB: heart rate response to deep breathing; BRS_VM: baroreflex sensitivity obtained by Valsalva maneuver; BRS_seq: baroreflex sensitivity obtained by the sequence method; LF: low frequency; HF: high frequency; FVC: forced vital capacity; FEV1: forced expiratory volume in one second; MIP: maximum inspiratory pressures; MEP: maximum expiratory pressures; RMT: respiratory muscle training. ^∗^Significant difference (*p* < 0.05) between follow-up and baseline.

**Table 3:** Comparison of MG outcomes and fatigue score before and after RMT.

	RMT group (*n* = 18)			Disease control group (*n* = 16)		
	Baseline	Follow-up	*p* value	Baseline	Follow-up	*p* value
Outcome measures						
QMG score	9.5 [5.5, 12.75]	7.5 [4, 12]	0.02^∗^	12.5 [4.75, 14.75]	9.5 [5.5, 13.25]	0.11
MGC scale	4 [1.25, 6.75]	2 [0.25, 3]	0.05^∗^	3.5 [0, 8]	3.5 [0.75, 4.75]	0.26
Fatigue scale						
MFSI_SF_general	7 [5.0, 10.75]	7 [6.0, 8.75]	0.54	6 [4.75, 8.25]	8 [5.75, 11.75]	0.21
MFSI_SF_physical	8 [5.25, 8.75]	5 [3.0, 8.0]	0.02^∗^	7 [4.75, 9.75]	8.5 [5.75, 11.75]	0.12
MFSI_SF_emotion	6 [3.25, 7.0]	4 [3, 8.75]	0.46	5 [3, 8.25]	7 [4, 8.25]	0.63
MFSI_SF_mental	6.5 [6.0, 8.0]	7 [3.5, 8.75]	0.87	6 [5.75, 9.5]	7.5 [6, 11]	0.29
MFSI_SF_vigor	10 [8.0, 13.75]	11.5 [10, 13]	0.16	15 [11.5, 15.75]	11 [6, 15.25]	0.50
MFSI_SF_total	14 [5, 23.5]	13 [1.25, 22]	0.04^∗^	9 [0.75, 25.75]	21.5 [6, 34.75]	0.09

QMG: quantitative myasthenia gravis; MGC: myasthenia gravis composite; MFSI-SF: Multidimensional Fatigue Symptom Inventory-Short Form; RMT: respiratory muscle training. ^∗^Significant difference (*p* ≤ 0.05) between follow-up and baseline.

## Data Availability

The data used to support the findings of this study are available from the corresponding author upon request.

## Abbreviations

FVC: Forced vital capacity
FEV1: Forced expiratory volume in one second
IQR: Interquartile range
MEP: Maximum expiratory pressures
MG: Myasthenia gravis
MGC: Myasthenia gravis composite
MGFA: Myasthenia Gravis Foundation of America
MIP: Maximum inspiratory pressures
MFSI-SF: Multidimensional Fatigue Symptom Inventory-Short Form
QMG: Quantitative myasthenia gravis
RMT: Respiratory muscle training
SD: Standard deviation
VC: vital capacity.

## References

[1] Berrih-Aknin S., Le Panse R. (2014). Myasthenia gravis: a comprehensive review of immune dysregulation and etiological mechanisms. *Journal of Autoimmunity*.

[2] Heliopoulos I., Patlakas G., Vadikolias K. (2003). Maximal voluntary ventilation in myasthenia gravis. *Muscle & Nerve*.

[3] Chaudhuri A., Behan P. O. (2009). Myasthenic crisis. *QJM*.

[4] Jaretzki A., Barohn R. J., Ernstoff R. M. (2000). Myasthenia gravis: recommendations for clinical research standards. Task Force of the Medical Scientific Advisory Board of the Myasthenia Gravis Foundation of America. *Neurology*.

[5] Rassler B., Marx G., Hallebach S., Kalischewski P., Baumann I. (2011). Long-term respiratory muscle endurance training in patients with myasthenia gravis: first results after four months of training. *Autoimmune Diseases*.

[6] Freitag S., Hallebach S., Baumann I., Kalischewski P., Rassler B. (2018). Effects of long-term respiratory muscle endurance training on respiratory and functional outcomes in patients with myasthenia gravis. *Respiratory Medicine*.

[7] Fregonezi G. A., Resqueti V. R., Guell R., Pradas J., Casan P. (2005). Effects of 8-week, interval-based inspiratory muscle training and breathing retraining in patients with generalized myasthenia gravis. *Chest*.

[8] Rassler B., Hallebach G., Kalischewski P., Baumann I., Schauer J., Spengler C. M. (2007). The effect of respiratory muscle endurance training in patients with myasthenia gravis. *Neuromuscular Disorders*.

[9] Berrih-Aknin S., Frenkian-Cuvelier M., Eymard B. (2014). Diagnostic and clinical classification of autoimmune myasthenia gravis. *Journal of Autoimmunity*.

[10] Forno E., Han Y. Y., Mullen J., Celedon J. C. (2018). Overweight, Obesity, and Lung Function in Children and Adults—A Meta-analysis. *The Journal of Allergy and Clinical Immunology: In Practice*.

[11] Chen H. I., Kuo C. S. (1989). Relationship between respiratory muscle function and age, sex, and other factors. *Journal of Applied Physiology*.

[12] Sa-Caputo D., Paineiras-Domingos L. L., Francisca-Santos A. (2019). Whole-body vibration improves the functional parameters of individuals with metabolic syndrome: an exploratory study. *BMC Endocrine Disorders*.

[13] Des Jarlais D. C., Lyles C., Crepaz N., Group T. (2004). Improving the reporting quality of nonrandomized evaluations of behavioral and public health interventions: the TREND statement. *American Journal of Public Health*.

[14] Keenan S. P., Alexander D., Road J. D., Ryan C. F., Oger J., Wilcox P. G. (1995). Ventilatory muscle strength and endurance in myasthenia gravis. *The European Respiratory Journal*.

[15] Sapienza C. M. (2008). Respiratory muscle strength training applications. *Current Opinion in Otolaryngology & Head and Neck Surgery*.

[16] Chen P. C., Liaw M. Y., Wang L. Y. (2016). Inspiratory muscle training in stroke patients with congestive heart failure: a CONSORT-compliant prospective randomized single-blind controlled trial. *Medicine*.

[17] Miller M. R., Crapo R., Hankinson J. (2005). General considerations for lung function testing. *European Respiratory Journal*.

[18] Johnson J. D., Theurer W. M. (2014). A stepwise approach to the interpretation of pulmonary function tests. *American Family Physician*.

[19] Andersen L. K., Knak K. L., Witting N., Vissing J. (2016). Two- and 6-minute walk tests assess walking capability equally in neuromuscular diseases. *Neurology*.

[20] Crescimanno G., Modica R., Lo Mauro R., Musumeci O., Toscano A., Marrone O. (2015). Role of the cardio-pulmonary exercise test and six-minute walking test in the evaluation of exercise performance in patients with late-onset Pompe disease. *Neuromuscular Disorders*.

[21] Gibbons W. J., Fruchter N., Sloan S., Levy R. D. (2001). Reference values for a multiple repetition 6-minute walk test in healthy adults older than 20 years. *Journal of Cardiopulmonary Rehabilitation*.

[22] Casanova C., Celli B. R., Barria P. (2011). The 6-min walk distance in healthy subjects: reference standards from seven countries. *European Respiratory Journal*.

[23] Muppidi S. (2017). Outcome measures in myasthenia gravis: incorporation into clinical practice. *Journal of Clinical Neuromuscular Disease*.

[24] Burns T. M., Conaway M., Sanders D. B. (2010). The MG composite: a valid and reliable outcome measure for myasthenia gravis. *Neurology*.

[25] Benatar M., Sanders D. B., Burns T. M. (2012). Recommendations for myasthenia gravis clinical trials. *Muscle & Nerve*.

[26] Barnett C., Herbelin L., Dimachkie M. M., Barohn R. J. (2018). Measuring clinical treatment response in myasthenia gravis. *Neurologic Clinics*.

[27] Pien L. C., Chu H., Chen W. C. (2011). Reliability and validity of a Chinese version of the multidimensional fatigue symptom inventory-short form (MFSI-SF-C). *Journal of Clinical Nursing*.

[28] Stein K. D., Jacobsen P. B., Blanchard C. M., Thors C. (2004). Further validation of the multidimensional fatigue symptom inventory-short form. *Journal of Pain and Symptom Management*.

[29] Weiner P., Gross D., Meiner Z. (1998). Respiratory muscle training in patients with moderate to severe myasthenia gravis. *Canadian Journal of Neurological Sciences*.

[30] Leith D. E., Bradley M. (1976). Ventilatory muscle strength and endurance training. *Journal of Applied Physiology*.

[31] Burns T. M. (2012). The MG composite: an outcome measure for myasthenia gravis for use in clinical trials and everyday practice. *Annals of the New York Academy of Sciences*.

[32] Alekseeva T. M., Gavrilov Y. V., Kreis O. A., Valko P. O., Weber K. P., Valko Y. (2018). Fatigue in patients with myasthenia gravis. *Journal of Neurology*.

[33] Tran C., Bril V., Katzberg H. D., Barnett C. (2018). Fatigue is a relevant outcome in patients with myasthenia gravis. *Muscle & Nerve*.

[34] McKenna M. J., Harmer A. R., Fraser S. F., Li J. L. (1996). Effects of training on potassium, calcium and hydrogen ion regulation in skeletal muscle and blood during exercise. *Acta Physiologica Scandinavica*.

[35] Lindinger M. I., McKelvie R. S., Heigenhauser G. J. (1995). K+ and Lac- distribution in humans during and after high-intensity exercise: role in muscle fatigue attenuation?. *Journal of Applied Physiology*.

